# Glioblastoma—a moving target

**DOI:** 10.3109/03009734.2012.676574

**Published:** 2012-04-19

**Authors:** Bengt Westermark

**Affiliations:** Uppsala University, Department of Immunology, Genetics and Pathology, Rudbeck Laboratory, SE-751 85 Uppsala, Sweden

**Keywords:** Brain tumors, molecular biology, oncogenes, pathogenesis, suppressor genes, tumor biology

## Abstract

The slow development of effective treatment of glioblastoma is contrasted by the rapidly advancing research on the molecular mechanisms underlying the disease. Amplification and overexpression of receptor tyrosine kinases, particularly EGFR and PDGFRA, are complemented by mutations in the PI3K, RB1, and p53 signaling pathways. In addition to finding effective means to target these pathways, we may take advantage of the recent understanding of the hierarchical structure of tumor cell populations, where the progressive expansion of the tumor relies on a minor subpopulation of glioma stem cells, or glioma-initiating cells. Finding ways to reprogram these cells and block their self-renewal is one of the most important topics for future research.

## Introduction

Glioblastoma is the most common intracranial malignancy and constitutes about 50% of all gliomas. With an annual incidence of 3–5 cases per 100,000 individuals, it is an uncommon type of malignancy, but its localization in the brain, its invasive behavior, and its extremely poor prognosis make it one of the most dreaded forms of cancer. The overall median survival time is as short as some 15 months despite the combined therapy of neurosurgery, radiation, and temozolomide (1). The slow development of an effective therapy is in bright contrast to the rapidly growing knowledge of the molecular pathogenesis of the disease.

Only a quarter of a century ago, molecular neuro-oncology was a rather neglected research field, which may be difficult to understand today when a great number of advanced neuro-oncology papers are published yearly, often in high-profile journals. In most cases, experimental research on glioblastoma in the old days was confined to a few dedicated and specialized centers under the leadership of neuropathologists, neuro-oncologists, and neurosurgeons. Today, research on glioblastoma is more widespread and often pursued by investigators with a broader interest in tumor biology and tumor genetics.

Permanent glioblastoma cell lines have been available since the mid-1960s through the work of Jan Pontén and myself (2,3). Additional glioblastoma cell lines were later established by other centers, yielding the Duke (D-) (4), Lausanne (LN-) (5), and University of California, San Francisco (SF-) (6) series of cell lines, in addition to the Uppsala (U-) series. Together, these ‘classical’ cell lines have contributed to our understanding of glioblastoma cell biology and, importantly, fueled our enthusiasm in molecular and cellular neuro-oncology. However, I think it is fair to say that the boom in brain tumor research during the late 1980s and 190s did not primarily stem from research on the phenotypic characteristics of cell lines. My personal view is that the field became attractive due to a few paradigmatic findings on brain tumor genetics, which placed brain tumors in the limelight. Some of these breakthroughs will be described below.

## Progress of the understanding of the molecular pathogenesis of glioblastoma

Although Stanley Cohen identified and purified epidermal growth factor (EGF) as early as 1962 (7), it was only after the discovery of the EGF receptor (8) that the scientific community in general became aware of the biological importance of this growth factor. In 1976, during my visit to Stanley Cohen's laboratory at Vanderbilt University, we showed that human glioma cell lines were endowed with EGF receptors in relatively high numbers (Westermark B, Carpenter G, and Cohen S, unpublished). A few years later we showed that one of the cell lines responded to EGF by a profound increase in cell motility (9). However, the breakthrough in the understanding of the importance of EGF in glioma biology came through Joseph Schlessinger and collaborators' studies on EGF receptor expression in glioma tissue (10). Subsequent to the cloning of the receptor cDNA, Schlessinger et al. demonstrated that the increased expression was due to *EGFR* gene amplification (11). Overexpression of wild-type or truncated and constitutively activated EGFR is now considered an important event in the pathogenesis of a subset of glioblastoma. The finding of a high frequency of *EGFR* amplification provided an explanation to the often occurring double minute chromosomes in glioblastoma (12); these are known to harbor amplified DNA segments. In addition to the impact on our understanding of the biology of glioblastoma, Schlessinger's seminal studies contributed to the increasing interest of the research community in this particular malignancy. Bert Vogelstein's contribution to the field is another example. In a survey of mutations in the TP53 gene, Vogelstein's research group found that glioblastoma was among those with the highest frequency of mutations. Vogelstein's cloning of *GLI1* from amplified DNA in glioblastoma was another highlight along the road (13).

Cytogenetic studies performed by Sandra Bigner and Joakim Mark and collaborators showed that loss of one copy of chromosome 10 is a common characteristic of glioblastoma (12). The search for a tumor suppressor gene on chromosome 10 made progress when *PTEN* was identified (14) and found to be frequently mutated in glioblastoma. As an important inhibitor along the phosphatidylinositol 3-kinase (PI3K) pathway, PTEN has attracted considerable general interest and made glioblastoma an interesting model for further studies.

Structural abnormality in the short arm of chromosome 9 is another common cytogenetic finding in glioblastoma. Mark Skolnick and collaborators highlighted the importance of this abnormality when they identified a tumor suppressor locus harboring the gene for the cell cycle regulators INK4A and ARF (15), which are key regulators of the RB1 and p53 pathways, respectively. Although Skolnic's work was primarily performed on melanomas, gliomas were also included in the study and found to have frequent deletions of the tumor suppressor locus.

My own work in the glioblastoma field was initiated during my graduate studies, when I established human cell lines and analyzed their growth behavior (3,16). These studies were the theme of my doctoral thesis in 1973 but also left me with considerable frustration, because of the phenotypic diversity of the cell lines and lack of molecular tools for mechanistic studies. Already in my very first publication (17) I became aware of the importance of serum-derived growth factors in growth regulation, thanks to the work of Holley and Kiernan (18). My simple and somewhat naive reasoning at this point was that in order to study seriously the deficient growth control of cancer cells there is a need for a better understanding of the growth regulation of normal cells. To do that, one needs to identify and mechanistically study factors that regulate cell proliferation. At that time, Howard Temin and others had proposed that transformed cells may stimulate their proliferation by their own growth factors, later known as autocrine growth stimulation (19). After initial studies on EGF and other growth factors, my colleagues Åke Wasteson, Carl-Henrik Heldin, and I focused on platelet-derived growth factor (PDGF) and its protein tyrosine kinase receptor. Parallel to our work on PDGF, we also characterized a growth factor produced by osteosarcoma cells. During the progress of this work, we became increasingly aware of the similarities of this growth factor and PDGF (20). Later, the osteosarcoma-derived growth factor was indeed shown to be a homodimer of PDGF A-chains (21), while the major part of PDGF purified from platelets is constituted by PDGF-AB.

During the rapid progress of the work on PDGF, I slowly lost interest in glioma biology, and at one point I decided to drop it entirely. Much influenced by our work on the osteosarcoma-derived growth factor and its putative role as an autocrine growth factor, I did one experiment which would bring me back to the glioblastoma research field. Conditioned medium from glioblastoma cell cultures was shown to contain a PDGF receptor-displacing activity, which through the work of Monica Nistér and others was shown to be identical to PDGF (22,23). A clonal derivative of the glioblastoma cell line U-343 MGa was shown to produce high amounts of PDGF-AA and was used by Christer Betsholtz and co-workers to clone A-chain cDNA (24). Correlative studies on human glioblastoma biopsies revealed the concomitant expression of PDGF α-receptors and PDGF A-chains (25), providing circumstantial evidence for an autocrine stimulation of glioma growth.

The finding that the v-*sis* oncogene of simian sarcoma virus (SSV) is a retroviral homolog of the PDGF B-chain gene (26,27) was paradigmatic in the sense that it helped us understand the molecular mechanisms of oncogene-driven cell transformation in relation to growth factor-induced cell proliferation (28). The finding also showed that we might be on the right track in our studies of PDGF influences on glioma development.

Most remarkably, Friedrich Deinhardt had previously found that SSV induced brain tumors similar to glioblastoma in newborn marmosets (29). This finding indicated that forced expression of PDGF in brain cells is indeed oncogenic and thus provided unequivocal evidence for the pathogenetic role of autocrine growth stimulation in gliomagenesis. However, given the widely adopted view that fully malignant tumors evolve through several genetic changes in a multistep and multistage process, it was not simple to envisage how a single growth factor-encoding gene could induce malignant brain tumors. We reasoned that the expression of the v-*sis* gene might be complemented by secondary changes in the genome, induced by proviral insertional mutagenesis (30). Using a PDGF-encoding Moloney mouse leukemia virus construct (31), Lene Uhrbom and Fredrik Johansson were able to identify a number of common proviral insertions in experimental mouse brain tumors, known oncogenes and suppressor genes as well as novel candidate genes (32). The functional role of a few of them has been studied by us and others (33–37).

The experimental evidence described above, in conjunction with the finding of PDGF α-receptor gene amplification in human glioblastoma (38), provides strong support for the role of PDGF as a driver of tumor growth in a glioblastoma subset. This view is strongly supported by analyses by the Cancer Genome Atlas Research Network (39). Three core pathways were identified, namely the receptor tyrosine kinase/RAS/phosphatidylinositol 3-kinase, p53, and RB signaling pathways. More recently, the network published evidence for the existence of four distinct types of glioblastoma: the classical, the mesenchymal, the neural, and the proneural subtype (40). The proneural subtype is characterized by aberrations in the PDGF α-receptor pathway, in addition to mutations in the isocitrate dehydrogenase 1 (*IDH1*) gene. The gliomagenic role of PDGF is further discussed in this issue (41–43).

## Development of future therapy

As mentioned above, the detailed dissection of the molecular biology of glioblastoma is in striking contrast to the lack of advances in therapy. If one looks at the problem from the bright side, a number of potential targets have been identified, and we now only need to find or develop potent inhibitors and use combinations of them in a clever way. There are, however, several hurdles along the road: 1) We do not have effective inhibitors (or agonists) against all identified targets. 2) Any effective molecule has to pass the blood–brain barrier in order to reach all invading cells, including those located at a distance from the center part of the tumor, the only part of the tumor where the barrier is disrupted. 3) We still lack the perfect animal model for treatment studies. A major problem is that the migrating cells constitute a major challenge and are literally a moving target. They blend with the normal tissue, are difficult to identify and target, and do not elicit an angiogenic response, making anti-angiogenesis treatment non-effective or even causing adverse effects; angiogenesis blockade may potentiate invasion (44). There are, however, other avenues for the development of effective glioma therapy which have not yet been fully exploited.

Only a decade ago, most of us had quite a simplistic view of the development of glioblastoma and other solid malignancies, much influenced by Peter Nowell's clonal evolution theory (45) and Bert Vogelstein's concept of sequential acquirement of mutations in tumor progression (46). These models proposed a non-hierarchical tumor cell population, in which any single cell at any given moment could give rise to a progeny replicating the malignant properties of the parental tumor, e.g. in the metastatic process. This view has been challenged by experiments that show that tumor growth may be fueled by a minor population of malignant cells with stem cell properties. Like stem cells, they can undergo self-renewal but also give rise to a clonal expansion of a rapidly growing progeny which will constitute the bulk of the tumor mass but have a limited replication potential. The former cells may re-initiate tumor growth, whereas the latter have lost this capacity (see (47) for an illuminating review). Experimental evidence for the presence of cancer stem cells in glioblastoma (or glioma-initiating cells (GICs)) has been provided by several authors (see (48) for a recent review). The origin of these cells is discussed in this issue, (41). In my view, the most interesting aspect of the cancer stem cell concept is that it opens up new avenues for the development of therapeutic strategies. The self-renewal capacity of the GICs can be modulated by extrinsic factors, in which members of the TGF-β superfamily play important roles. Kohei Miyazono and collaborators have shown that the proliferation GICs is sustained by TGF-β, with Sox4 and Sox2/Oct4 as downstream effectors (49,50). Blocking the TGF-β pathway leads to growth inhibition and induces differentiation. Moreover, BMP4 has been shown to be a GIC antagonist; pretreatment *in vitro* of glioblastoma cells inhibits their tumor-initiating capacity after injection of the cytokine (51). We have also shown that growth inhibition can be induced by forced expression of Sox21 (52), which is a Sox2 antagonist (53). Thus, despite all the mutations leading to aberrant signaling in glioblastoma described above, the cells are not entirely refractory to differentiation-inducing and cell cycle-blocking signals. Identification of small molecules that pass the blood–brain barrier and target these signals seems to be one of the most important goals in the search for future therapy.

When I as a young scientist tried to establish human glioblastoma cell lines, I found to my disappointment that only some 20% of the tumor biopsies gave rise to permanent cell lines (3). In the remaining 80% of the cases, cells were able to divide and survive in primary culture only. We know today that the progressive growth of glioblastoma cells requires other and more sophisticated culture conditions (growth factor-containing, serum-free neural stem cell medium) than we used in the past (Eagle's minimum essential medium with 10% calf serum). Bovine serum contains BMP4 (54) and perhaps other, yet unidentified, factors which may have caused the apparent irreversible growth inhibition that we observed. I used to say that the best way to inhibit the growth of glioblastoma cells is to put them into a culture dish and feed them with 10% serum. The same finding that made me disappointed 40 years ago makes me now look at the future development of research on glioblastoma with great optimism.

## References

[1] Preusser M, de Ribaupierre S, Wohrer A, Erridge SC, Hegi M, Weller M (2011). Current concepts and management of glioblastoma. Ann Neurol.

[2] Ponten J, Macintyre EH (1968). Long term culture of normal and neoplastic human glia. Acta Pathol Microbiol Scand.

[3] Westermark B, Ponten J, Hugosson R (1973). Determinants for the establishment of permanent tissue culture lines from human gliomas. Acta Pathol Microbiol Scand A.

[4] Bigner DD, Bigner SH, Ponten J, Westermark B, Mahaley MS, Ruoslahti E (1981). Heterogeneity of genotypic and phenotypic characteristics of fifteen permanent cell lines derived from human gliomas. J Neuropathol Exp Neurol.

[5] Studer A, de Tribolet N, Diserens AC, Gaide AC, Matthieu JM, Carrel S (1985). Characterization of four human malignant glioma cell lines. Acta Neuropathol.

[6] Rutka JT, Giblin JR, Dougherty DY, Liu HC, McCulloch JR, Bell CW (1987). Establishment and characterization of five cell lines derived from human malignant gliomas. Acta Neuropathol.

[7] Cohen S (1962). Isolation of a mouse submaxillary gland protein accelerating incisor eruption and eyelid opening in the new-born animal. J Biol Chem.

[8] Carpenter G, Lembach KJ, Morrison MM, Cohen S (1975). Characterization of the binding of 125-I-labeled epidermal growth factor to human fibroblasts. J Biol Chem.

[9] Westermark B, Magnusson A, Heldin CH (1982). Effect of epidermal growth factor on membrane motility and cell locomotion in cultures of human clonal glioma cells. J Neurosci Res.

[10] Libermann TA, Razon N, Bartal AD, Yarden Y, Schlessinger J, Soreq H (1984). Expression of epidermal growth factor receptors in human brain tumors. Cancer Res.

[11] Libermann TA, Nusbaum HR, Razon N, Kris R, Lax I, Soreq H (1985). Amplification, enhanced expression and possible rearrangement of EGF receptor gene in primary human brain tumours of glial origin. Nature.

[12] Bigner SH, Mark J, Burger PC, Mahaley MS, Bullard DE, Muhlbaier LH (1988). Specific chromosomal abnormalities in malignant human gliomas. Cancer Res.

[13] Kinzler KW, Bigner SH, Bigner DD, Trent JM, Law ML, O'Brien SJ (1987). Identification of an amplified, highly expressed gene in a human glioma. Science.

[14] Li J, Yen C, Liaw D, Podsypanina K, Bose S, Wang SI (1997). PTEN, a putative protein tyrosine phosphatase gene mutated in human brain, breast, and prostate cancer. Science.

[15] Kamb A, Gruis NA, Weaver-Feldhaus J, Liu Q, Harshman K, Tavtigian SV (1994). A cell cycle regulator potentially involved in genesis of many tumor types. Science.

[16] Westermark B (1973). The deficient density-dependent growth control of human malignant glioma cells and virus-transformed glia-like cells in culture. Int J Cancer.

[17] Ponten J, Westermark B, Hugosson R (1969). Regulation of proliferation and movement of human glialike cells in culture. Exp Cell Res.

[18] Holley RW, Kiernan JA (1968). "Contact inhibition" of cell division in 3T3 cells. Proc Natl Acad Sci USA.

[19] Sporn MB, Todaro GJ (1980). Autocrine secretion and malignant transformation of cells. N Engl J Med.

[20] Heldin CH, Westermark B, Wasteson A (1980). Chemical and biological properties of a growth factor from human-cultured osteosarcoma cells: resemblance with platelet-derived growth factor. J Cell Physiol.

[21] Heldin CH, Johnsson A, Wennergren S, Wernstedt C, Betsholtz C, Westermark B (1986). A human osteosarcoma cell line secretes a growth factor structurally related to a homodimer of PDGF A-chains. Nature.

[22] Nister M, Hammacher A, Mellstrom K, Siegbahn A, Ronnstrand L, Westermark B (1988). A glioma-derived PDGF A chain homodimer has different functional activities from a PDGF AB heterodimer purified from human platelets. Cell.

[23] Hammacher A, Nister M, Westermark B, Heldin CH (1988). A human glioma cell line secretes three structurally and functionally different dimeric forms of platelet-derived growth factor. Eur J Biochem.

[24] Betsholtz C, Johnsson A, Heldin CH, Westermark B, Lind P, Urdea MS (1986). cDNA sequence and chromosomal localization of human platelet-derived growth factor A-chain and its expression in tumour cell lines. Nature.

[25] Hermanson M, Funa K, Hartman M, Claesson-Welsh L, Heldin CH, Westermark B (1992). Platelet-derived growth factor and its receptors in human glioma tissue: expression of messenger RNA and protein suggests the presence of autocrine and paracrine loops. Cancer Res.

[26] Doolittle RF, Hunkapiller MW, Hood LE, Devare SG, Robbins KC, Aaronson SA (1983). Simian sarcoma virus onc gene, v-sis, is derived from the gene (or genes) encoding a platelet-derived growth factor. Science.

[27] Waterfield MD, Scrace GT, Whittle N, Stroobant P, Johnsson A, Wasteson A (1983). Platelet-derived growth factor is structurally related to the putative transforming protein p28sis of simian sarcoma virus. Nature.

[28] Heldin CH, Westermark B (1984). Growth factors: mechanism of action and relation to oncogenes. Cell.

[29] Deinhardt F (1980). Biology of primate retroviruses.

[30] Westermark B, Johnsson A, Betsholtz C, Heldin CH (1987). Biological properties of simian sarcoma virus and its oncogene product.

[31] Uhrbom L, Hesselager G, Nister M, Westermark B (1998). Induction of brain tumors in mice using a recombinant platelet-derived growth factor B-chain retrovirus. Cancer Res.

[32] Johansson FK, Brodd J, Eklof C, Ferletta M, Hesselager G, Tiger CF (2004). Identification of candidate cancer-causing genes in mouse brain tumors by retroviral tagging. Proc Natl Acad Sci USA.

[33] Ferletta M, Uhrbom L, Olofsson T, Ponten F, Westermark B (2007). Sox10 has a broad expression pattern in gliomas and enhances platelet-derived growth factor-B–induced gliomagenesis. Mol Cancer Res.

[34] Tchougounova E, Jiang Y, Brasater D, Lindberg N, Kastemar M, Asplund A (2009). Sox5 can suppress platelet-derived growth factor B-induced glioma development in Ink4a-deficient mice through induction of acute cellular senescence. Oncogene.

[35] Westermark UK, Lindberg N, Roswall P, Brasater D, Helgadottir HR, Hede SM (2011). RAD51 can inhibit PDGF-B-induced gliomagenesis and genomic instability. Neuro Oncol.

[36] Wolf RM, Draghi N, Liang X, Dai C, Uhrbom L, Eklof C (2003). p190RhoGAP can act to inhibit PDGF-induced gliomas in mice: a putative tumor suppressor encoded on human chromosome 19q13.3. Genes Dev.

[37] Phillips JJ, Huillard E, Robinson AE, Ward A, Lum DH, Polley MY (2012). Heparan sulfate sulfatase SULF2 regulates PDGFRalpha signaling and growth in human and mouse malignant glioma. J Clin Invest.

[38] Fleming TP, Saxena A, Clark WC, Robertson JT, Oldfield EH, Aaronson SA (1992). Amplification and/or overexpression of platelet-derived growth factor receptors and epidermal growth factor receptor in human glial tumors. Cancer Res.

[39] Cancer Genome Atlas Research Network (2008). Comprehensive genomic characterization defines human glioblastoma genes and core pathways. Nature.

[40] Verhaak RG, Hoadley KA, Purdom E, Wang V, Qi Y, Wilkerson MD (2010). Integrated genomic analysis identifies clinically relevant subtypes of glioblastoma characterized by abnormalities in PDGFRA, IDH1, EGFR, and NF1. Cancer Cell.

[41] Jiang Y, Uhrbom L (2012). On the origin of glioma. Ups J Med Sci.

[42] Lindberg N, Holland EC (2012). PDGF in gliomas: more than just a growth factor?. Ups J Med Sci.

[43] Nazarenko I, Hede S-M, He X, Hedrén A, Thompson J, Lindström MS (2012). PDGF and PDGF receptors in glioma. Ups J Med Sci.

[44] Keunen O, Johansson M, Oudin A, Sanzey M, Rahim SA, Fack F (2011). Anti-VEGF treatment reduces blood supply and increases tumor cell invasion in glioblastoma. Proc Natl Acad Sci USA.

[45] Nowell PC (1976). The clonal evolution of tumor cell populations. Science.

[46] Vogelstein B, Fearon ER, Hamilton SR, Kern SE, Preisinger AC, Leppert M (1988). Genetic alterations during colorectal-tumor development. N Engl J Med.

[47] Gupta PB, Chaffer CL, Weinberg RA (2009). Cancer stem cells: mirage or reality?. Nat Med.

[48] Venere M, Fine HA, Dirks PB, Rich JN (2011). Cancer stem cells in gliomas: identifying and understanding the apex cell in cancer's hierarchy. Glia.

[49] Ikushima H, Todo T, Ino Y, Takahashi M, Miyazawa K, Miyazono K (2009). Autocrine TGF-beta signaling maintains tumorigenicity of glioma-initiating cells through Sry-related HMG-box factors. Cell Stem Cell.

[50] Ikushima H, Todo T, Ino Y, Takahashi M, Saito N, Miyazawa K (2011). Glioma-initiating cells retain their tumorigenicity through integration of the Sox axis and Oct4 protein. J Biol Chem.

[51] Piccirillo SG, Reynolds BA, Zanetti N, Lamorte G, Binda E, Broggi G (2006). Bone morphogenetic proteins inhibit the tumorigenic potential of human brain tumour-initiating cells. Nature.

[52] Ferletta M, Caglayan D, Mokvist L, Jiang Y, Kastemar M, Uhrbom L (2011). Forced expression of Sox21 inhibits Sox2 and induces apoptosis in human glioma cells. Int J Cancer.

[53] Sandberg M, Kallstrom M, Muhr J (2005). Sox21 promotes the progression of vertebrate neurogenesis. Nat Neurosci.

[54] Herrera B, Inman GJ (2009). A rapid and sensitive bioassay for the simultaneous measurement of multiple bone morphogenetic proteins. Identification and quantification of BMP4, BMP6 and BMP9 in bovine and human serum. BMC Cell Biol.

